# ER Stress Activates NF-κB by Integrating Functions of Basal IKK Activity, IRE1 and PERK

**DOI:** 10.1371/journal.pone.0045078

**Published:** 2012-10-26

**Authors:** Arvin B. Tam, Ellen L. Mercado, Alexander Hoffmann, Maho Niwa

**Affiliations:** 1 Division of Biological Sciences, Section of Molecular Biology, University of California, San Diego, La Jolla, California, United States of America; 2 Department of Chemistry & Biochemistry, University of California San Diego, La Jolla, California, United States of America; University Health Network, Canada

## Abstract

NF-κB, a transcription factor, becomes activated during the Unfolded Protein Response (UPR), an endoplasmic reticulum (ER) stress response pathway. NF-κB is normally held inactive by its inhibitor, IκBα. Multiple cellular pathways activate IKK (IκBα Kinase) which phosphorylate IκBα leading to its degradation and NF-κB activation. Here, we find that IKK is required for maximum activation of NF-κB in response to ER stress. However, unlike canonical NFκB activation, IKK activity does not increase during ER stress, but rather the level of basal IKK activity is critical for determining the extent of NF-κB activation. Furthermore, a key UPR initiator, IRE1, acts to maintain IKK basal activity through IRE1's kinase, but not RNase, activity. Inputs from IRE1 and IKK, in combination with translation repression by PERK, another UPR initiator, lead to maximal NF-κB activation during the UPR. These interdependencies have a significant impact in cancer cells with elevated IKK/NF-κB activity such as renal cell carcinoma cells (786-0). Inhibition of IKK by an IKK inhibitor, which significantly decreases NF-κB activity, is overridden by UPR induction, arguing for the importance of considering UPR activation in cancer treatment.

## Introduction

After emerging from the ribosome, secreted and membrane proteins are initially targeted and translocated into the ER as nascent polypeptides. To ensure their proper function, these polypeptides will have to be folded into specific conformations and modified properly within the lumen of the ER. As production of unfolded or partially folded proteins will cause deliterious effects, the ER has a quality control mechanism to ensure only properly folded protiens can leave the ER. In response to increased demands of producing secreted or membrane proteins, collectively termed ‘ER stress,’ the ER functional capacity is adjusted by a signal transduction pathway called the Unfolded Protein Response (UPR). Activation of the UPR leads to not only changes in the transcription profile, but also global translation repression. These events together allow adjustment of overall ER functions [1], [2], [3]. Ultimately, cells that fail to re-establish the proper ER protein folding capacity will be eliminated by induction of apoptosis. UPR signaling is emerging as a contributing factor to the pathology of several human diseases including diabetes and cancer [4], [5], [6]. The UPR contributes to the growth and survival of tumors, and tumor microenvironments have been found to induce UPR signaling [4], [5].

In higher eukaryotes, UPR signaling is initiated by three ER transmembrane sensors, the kinase PERK, the kinase/RNase IRE1, and the transcription factor ATF6 (reviewed in [1], [2], [3]). Activation of PERK leads to global protein translation inhibition by phosphorylation of eIF2α, α translation initiation factor [7]. At the same time, PERK also promotes transcription of UPR-specific genes by increasing translation of the transcription factor ATF4 [7]. IRE1 excises an intron from XBP1 mRNA [8], generating a spliced version of mRNA coding for a more potent form of a UPR transcription factor. The third UPR sensor, ATF6, is an ER transmembrane protein with a transcription activation domain on its cytoplasmic side. In response to ER stress, ATF6 undergoes proteolysis within the transmembrane domain to release its cytoplasmic transactivation domain from the ER membrane, allowing it to enter into the nucleus [9]. Thus, each ER proximal sensor is ultimately responsible for activation of a transcription factor. Activation of ATF6, ATF4, and XBP1 by the UPR result in a complex pattern of gene regulation [2], [3]. UPR signaling aims to alleviate the high levels of misfolded proteins in the ER by increasing protein folding capacity through up-regulation of ER chaperones such as BiP, GRP94, calreticulin, and Erdj4 [10], [11]. If proper protein folding capacity in the ER cannot be restored, the UPR up-regulates genes such as CHOP that result in activation of apoptotic pathways. [10]

In addition, activation of the highly studied transcription factor NF-κB has been reported to be a consequence of ER stress [12], [13], [14], although its role during the UPR has yet to be determined. NFκB consists of a family of dimer forming transcription factors that include RelA(p65), p50, p52, RelB, and c-Rel with RelA(p65)/p50 being the canonical form. Normally held in the cytoplasm in complex with IκBα, an inhibitor of NF-κB, canonical activation of NF-κB involves phosphorylation of IκBα by IκB kinase (IKK), followed by proteasome-mediated degradation of IκBα. IKK is comprised of IKKα, IKKβ, and IKKγ (NEMO) subunits, and during canonical activation, IKKβ phosphorylates IκBα on serines at position 32 and 36, leading to its polyubiquitination and proteosomal degradation [15]. This frees NF-κB for nuclear accumulation, binding to consensus κB promoter sites, and transcriptional activation of target genes. Genes regulated by NF-κB primarily promote survival, making NF-κB a key player in the development of invasive tumors and metastases, and in resistance to certain chemotherapeutic agents [16].

NF-κB can be activated by several stimuli including inflammatory signals such as tumor necrosis factor alpha (TNFα), interleukin-1 (IL-1), lipopolysaccharide (LPS), and internal cell stresses such as DNA damage [17]. Signals initiated by these stimuli converge by activating the IKK complex resulting in IKK mediated degradation of IκBα. Thus, IKK activation is a key regulatory step of NF-κB activation. Recent reports reveal that the mechanism of NF-κB activation during ER stress may differ from this conventional activation. Specifically, studies linking NF-κB activation to PERK-mediated phosphorylation of eIF2α proposed NF-κB activation during ER stress was independent of IKK activation since IκBα phosphorylation was not detected in mouse embryonic fibroblasts (MEFs) [12], [13]. However, a rapid reduction in IκBα takes place via translation inhibition induced by eIF2α phosphorylation. Since IκBα has a short half life, this consequently results in an increase in free NF-κB, leading to its activation.

More recently, another report using a human breast cancer cell line found IRE1 physically interacts with IKK upon ER stress induction, leading to an increase in P-IκBα and the concomitant decrease in total IκBα levels, resulting in NF-κB activation. Furthermore, the initial activation of NF-κB led to production of TNFα leading to further cannonical NF-κB acitvation, ultimately leading to cell death [18]. Currently, the relationships between these reports, specifically IKK phosphorylation of IκBα during ER stress, are not clear, as different cell lines were used for their experiments. IKK is a master regulator of NF-κB activation, and signaling through IKK is critical for almost all NF-κB activating pathways. Therefore, understanding the involvement of IKK and its relationship to both IRE1 and PERK is critical for determining how NF-κB is activated during ER stress.

Using mouse embryonic fibroblasts (MEFs) knocked out for components of the pathway, we identified the individual roles of IRE1, PERK, and IKK, and showed that full activation of NF-κB druing ER stress requires contributions from all three components. Interestingly, we show that IKKβ is required for NF-κB activation, however no increase in IKK activity is seen during ER stress. This seemingly paradoxical result led us to the discovery that *basal* IKK kinase activity is sufficient and necessary for NF-κB signalling during ER stress. Furthermore, while PERK mediated translational inhibition is sufficient to activate NF-κB, cells lacking IRE1 cannot fully activate NF-κB due to decreased basal IKK activity. Also, increasing basal IKK activity in cells lacking IRE1 rescues NF-κB activity. Thus, basal IKK activity, maintained by IRE1, and PERK mediated translation inhibition work together to activate NF-κB during ER stress. Finally, we demonstrate a functional implication for this observation using cancer cells that have increased basal IKK activity where induction of the UPR can reverse the action of an IKK inhibiting drug.

## Results

### IKK is Required for Activation of NF-κB during UPR

**Figure 1:**
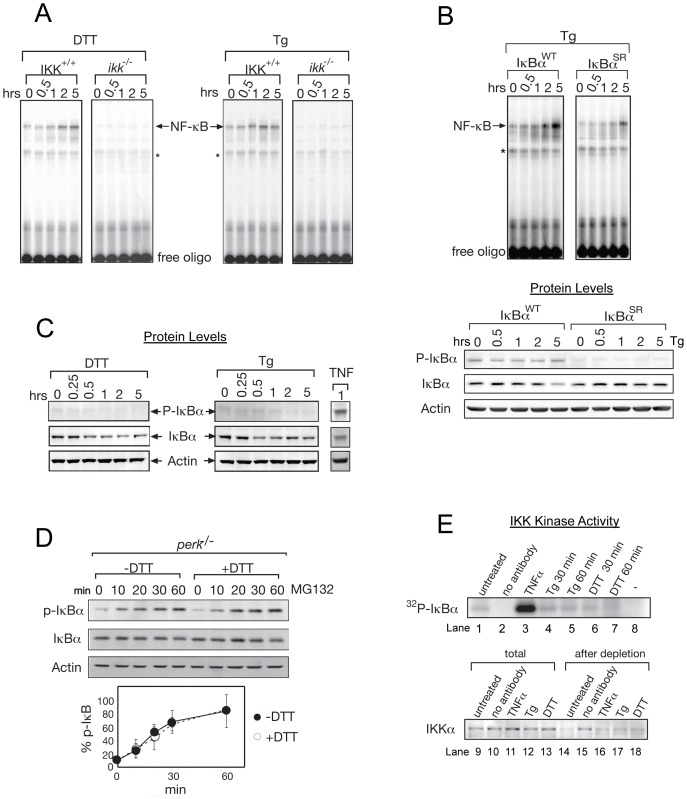
Basal IKK activity is required for NF-κB activation during ER stress. (A) NF-κB cannot be activated in the absence of IKK. Wild type MEFs (*IKK* ^+/+^) and MEFs knockedout for both IKKα and IKKβ (*ikk* ^−/−^) were treated with DTT or Tg, and EMSA was performed using a radiolabelled probe containing an NF-κB binding site (free oligo). ‘*’ denotes a background band (Figure S1) and free oligo shows that NF-κB probe was not limited for this assay. (B) IκBα phosphorylation is required for full NF-κB activation during ER stress. IκBα^−/−^ cells were reconstituted with either wild type IκBα (IκBα WT) or a non-phosphorylatable IκBα (IκBα SR) with Serines 32/36 were mutated to Alanines and were treated with Tg. NF-κB activation was analyzed by EMSA. Bottom Panel: Western blots against P-IκBα, total IκBα, and actin. IκBα was expressed equivalently in both cell types, and phosphorylation was only detected in IκBα WT but not IκBα SR. (C) IκBα phosphorylation does not increase during ER stress. Protein levels of phosphorylated IκBα (P-IκBα), total IκBα, and actin (loading control) were analyzed by western blot in WT MEFs treated wih DTT, Tg, or TNFα for indicated lengths of time (hrs). No increase in P-IκBα was detected, but a IκBα levels decreased. (D) IRE1 does not contribute to an increase in IKK activity during the UPR. In order to prevent the effect of the decreased IκBα levels due to translation inhibition, we used *perk* ^−/−^ MEFs. Cells were treated with MG132 in the absence (−DTT, closed circle) or presence (+DTT, open circle) of UPR induction. Levels of P-IκBα, total IκBα, and actin were determined by western blot. Accumulation of P-IκBα normalized to total IκBα was determined as a measure of basal IKK activity. At least, three independent experiments are performed to calculate standard error. (E) IKK kinase activity does not increase during ER stress. IKK kinase assay using IP'd IKK complexes from WT MEFs treated with either Tg, DTT or TNFα. IP'd complexes were incubated with recombinant IκBα and γ^32^P-ATP. Radiolabelled IκBα was then detected by autoradiography. Efficiencies of IKK IP were shown by depletion of IKKα (bottom panel). Total extracts and extracts after depletion were probed for IKKα protein levels to determine the efficiency of IKK complex IP. *No antibody* denotes a negative control using no antibody during IP (lanes 2, 10, 15). (−) shows a negative control where no kinase was added (lane 8).

To probe the mechanistic details of NF-κB activation during ER stress, in particular, potential involvement of IKK-mediated IκBα phosphorylation, we examined ER stress induced NF-κB activation in wild type (WT) mouse embryonic fibroblasts (MEFs) and MEFs lacking the α and β subunits of IKK (*ikk^−/−^*) [19], [20]. ER stress was induced by DTT and Thapsigargin (Tg), two well established UPR activating agents. DTT disrupts disulfide bonds resulting in unfolded proteins, while Tg ultimately blocks ER chaperone function by disrupting ER calcium levels. NF-κB was measured by electrophoretic mobility shift assay (EMSA) [14], which involves incubation of a radio labeled DNA probe containing the NF-κB binding site with nuclear extracts prepared from WT MEFs treated with DTT or Tg. We found that UPR induction resulted in NF-κB activation (Figure 1A, IKK^+/+^). Both competition and supershift assays confirmed that the activated form of NF-κB is the cannonical p65/p50 form (Figure S1A–S1C). Furthermore, consistent with the EMSA results, immunofluorescence detected NF-κB translocation into the nucleus during ER stress (Figure S1D). NF-κB activation was also observed with DTT and Tg in multiple cell types (Figure S2A–S2D). While wild type MEFs showed robust activation of NF-κB in different cell types, we found that NF-κB activation was significantly diminished in *ikk^−/−^* MEFs (Figure 1A, *ikk^−/−^*), indicating a requirement for IKK during UPR induced activation of NF-κB. To further determine which IKK subunit is required for NF-κB activation, we used MEFs lacking either *ikk*α*^−/−^* or *ikk*β*^−/−^* (Figure S1E). We found that cells lacking IKKα still induced NF-κB upon ER stress induction while cells lacking IKKβ were not able to induce NF-κB. Furthermore, we found that the kinase activity of IKKβ was required, as only kinase active IKKβ, not kinase dead IKKβ, can rescue NF-κB activity in *ikk*β*^−/−^* cells (Figure S1E). These results revealed the importance of IKKβ during ER stress, a similar requirement for cannonical NF-κB activation.

To further test involvement of IKK, we examined the requirement of IκBα phosphorylation. We took IκBα^−/−^ cells and added back either wild type IκBα (IκBα-WT) or a non-phosphorylatable IκBα where the IKK phosphorylation sites (Ser32/36) were mutated to Alanine (IκBα-SR), preventing its phosphorylation (Figure 1B lower panel, P- IκBα) [21]. Cells expressing IκBα-SR showed significantly reduced NF-κB activation during the UPR (Figure 1B upper panel). As IκBα was expressed at similar levels between cells expressing IκBα-WT and IκBα-SR (Figure 1B lower panels, IκBα), an inability to activate NF-κB in cells expressed IκBα-SR was not due to difference in its expression level from that of WT IκBα. Thus, together with diminished NF-κB activation in *ikk^−/−^* or *ikk*β*^−/−^* ^`^ MEFs (Figure 1A & S1E), these data suggested the importance of IKK mediated IκBα phosphorylation in UPR induced NF-κB activation.

During canonical NF-κB activation, IκBα phosphorylation leads to decreased IκBα levels due to proteasomal degradation. Similarly, total IκBα was decreased after UPR induction (Figure 1C, IκBα). Curiously, however, we found that phosphorylation of IκBα was not induced during the UPR time course (Figure 1C, P- IκBα), while canonical NF-κB activating agent, TNFα treatment clearly increased P- IκBα as expected. These results were replicated in another cell type (CHO) (Figure S2E). Since we did not detect increased phosphorylation of IκBα during ER stress, we wanted to further investigate whether the rate of IκBα phosphorylation changed during ER stress. Since phosphorylation of IκBα causes proteasome mediated degradation [22], we added a proteasome inhibitor, MG132, to *perk^−/−^* cells in order to block proteasomal degradation and measured the rate of accumulation of P- IκBα throughout the time course of ER stress (+DTT) or without (−DTT) ER stress (Figure 1D). It should be noted that these experiments were performed in *perk^−/−^* MEFs to avoid any change in IκBα levels caused by PERK induced translation repression in UPR induced cells, so that the rate of IκBα phosphorylation or IKK activity can be assessed. Rates of accumulation of P- IκBα in the presence of MG132 were equivalent in untreated and UPR-induced cells (Figure 1D), indicating that UPR activation does not change either the rate of IκBα phosphorylation or IKK activity. Therefore, UPR decreased the overall level of IκBα even though no significant increase in its phosphorylation was observed.

To further investigate these apparent discrepancies in the IKK involvement in NF-κB activation during UPR, we examined IKK kinase activity using immunoprecipitated (IP) IKK from total cell extracts, followed by incubation with recombinant IκBα substrate and (γ^32^P)-ATP (Figure 1E, top panel) using *in vitro* kinase assay. Efficient IP of the IKK complex occurred as seen by depletion of IKKα levels from extracts (Figure 1E, bottom panel, compare lanes 9–13 (total) with lanes 14–18 (after depletion)). TNFα showed high IKK kinase activity (Figure 1E, lane 3) [22], while UPR-induced cells showed IKK activity equal to uninduced levels (Figure 1E, lanes 1 and 4–7), indicating that IKK activity did not increase during ER stress. Taken together, our results revealed that IKK mediated IκBα phosphorylation was required for NFκ-B activation (Figure 1A & 1B), despite no increase in IKK activity or phosphorylaton of IκBα during the UPR (Figure 1C, 1D & 1E).

### PERK-Mediated Translation Repression is Not Sufficient to Fully Activate NF-κB During UPR in Cells Lacking IRE1

**Figure 2:**
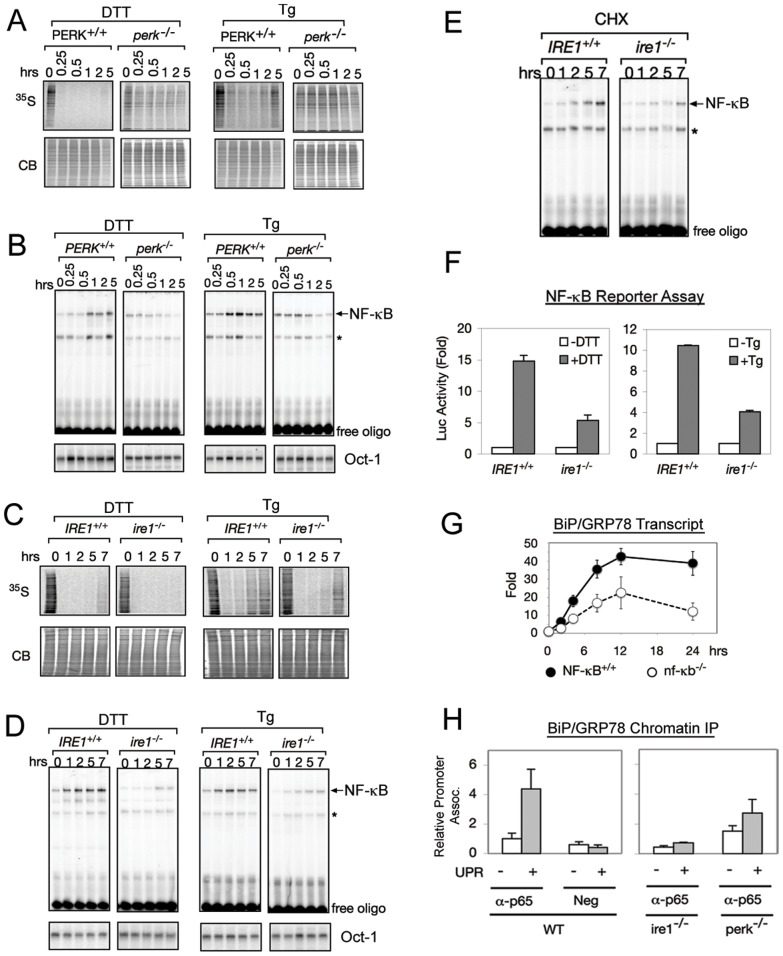
Translation inhibition is not sufficient to activate NF-κB during ER stress in the absence of IRE1. (A) PERK represses translation. PERK^+/+^ and *perk* ^−/−^ cells were treated with either DTT or Tg and pulsed with ^35^S-Met/Cys to measure level of translation (^35^S panel). Coomassie Blue (CB) staining showed equal levels of total protein. (B) PERK is required for ER stress induced NF-κB. EMSAs of PERK^+/+^ and *perk* ^−/−^ cells treated with either DTT or Tg are shown. Oct-1 EMSA assays were performed on the same nuclear extracts as a control. ‘*’ denotes a background band. (C) PERK dependent translation repression is normal in *ire1* ^−/−^ cells. *IRE1* ^+/+^ and *ire1* ^−/−^ cells were treated with DTT or Tg and pulsed with ^35^S-Met/Cys to measure level of translation (^35^S panel). Coomassie Blue (CB) staining showed equal levels of total protein. (D) IRE1 is necessary for full activation of NF-κB by ER stress. *IRE1* ^+/+^ and *ire1* ^−/−^ cells were treated with DTT or Tg, and NF-κB activation was determined by EMSA. Oct-1 EMSAs were performed as a control. ‘*’ denotes a background band. (E) IRE1 is necessary for NF-κB activation during translation inhibition. *IRE1* ^+/+^ and *ire1^−/−^* MEFs were treated with CHX and NF-κB activity was analyzed by EMSA. (F) IRE1 is necessary for full transcriptional activity of NF-κB during ER stress. *IRE1* ^+/+^ or *ire1* ^−/−^ cells were transfected with a 3X κB promoter fused to luciferase and treated with DTT or Tg. Luciferase reporter activity was measured as a readout of NF-κB activity. Fold change in luciferase activity in comparison to untreated cells is shown. (G) NF-κB is required for full induction of BiP transcript during the UPR. MEFs knocked out for p65 (nf-kb^−/−^, open circle) or WT cells (closed circle) were treated with Tg for 24 hrs and BiP mRNA was measured by real time PCR. Fold change in transcript levels in comparison to untreated cells is shown. (H) NF-κB binds to the BiP promoter. Chomatin IP was done on WT, ire1^−/−^, or perk^−/−^ cells using α-p65 antibody, and primers against the BiP promoter were used to amplify the IP'd fraction. Fold change in relative promoter association is shown in comparison to untreated wild type cells. Neg designates a negative control where no antibody was added to the ChIP. For all experiments in this figure, at least three independent experiments were performed to calculate standard error.

Another puzzling observation is the relative contribution of IRE1 and PERK. To date, there is no direct molecular event reported that simultaneously requires both IRE1 and PERK for its activation. To identify functional contributions of each component, we used MEFs knocked out in either IRE1 (*ire1* ^−/−^) or PERK (*perk* ^−/−^). Previous studies reported that PERK mediated translational inhibition was sufficient to induce NF-κB during the UPR [12]. Consequently, *perk^−/−^* MEFs which cannot mount UPR-induced translation inhibition (Figure 2A), resulted in significantly diminished activation of NF-κB during UPR activation when compared with WT (Figure 2B), in agreement with previous reports [12]. Furthermore, we found that the extent of translation inhibition determines the level of NF-κB activation (Figure S3A) in wild type cells. This indicates that the contribution of PERK, translation inhibition, is sufficient to activate NF-κB in wild type cells.

To determine the functional role of IRE1, we asked if PERK mediated translation inhibition was sufficient to activate NF-κB in cells lacking IRE1. We first determined that PERK mediated translation inhibition is equivalent in WT and *ire1* ^−/−^ cells (Figure 2C). If translation repression mediated reduction of IκBα was sufficient for maximum NF-κB activation during UPR, we anticipated NF-κB activation in *ire1* ^−/−^ cells to take place normally during ER stress, similar to that in WT cells. However, despite normal PERK-dependent translational inhibition, we found that the overall NF-κB activity was greatly diminished in *ire1^−/−^* cells (Figure 2D). Additionally, we used cycloheximide (CHX), a pharmacological inhibitor of translation, to substitute for PERK mediated translation inhibition [7], and found that CHX activated NF-κB in WT (*IRE1^+/+^*) cells, while CHX treatment of *ire1^−/−^* cells had significantly reduced NF-κB (Figure 2E). Taken together, these data demonstrated a functional contribution of IRE1 to NF-κB activation beyond PERK dependent translation inhibition. To further confirm that IRE1 does have a functional contribution, NF-κB activity in *ire1^−/−^* cells was restored by transfection of IRE1, where the extent of NF-κB recovery after IRE1 transfection was directly proportional to that of IRE1-mediated XBP1 splicing (Figure S3B & S3C). Together, these results showed that loss of either IRE1 or PERK resulted in diminished NF-κB signaling during the UPR, suggesting combined inputs from the IRE1 and PERK signaling branches were needed for NF-κB activation.

The importance of both IRE1 and PERK for NF-κB activation during the UPR was also evaluated at the downstream transcription level. Using an NF-κB luciferase reporter assay, reduced levels of luciferase was detected in *ire1* ^−/−^ cells (Figure 2F), in agreement with the reduced level of activated NF-κB binding to the DNA detected by EMSA (Figure 2D). To further test the functional significance of NF-κB activation during the UPR, we examined the induction of a UPR target gene, BiP/GRP78 in MEFs knocked out for p65 (nf-κb^−/−^). These cells did not show active NF-κB (data not shown). We found that in p65 knockout cells, BiP/GRP78 expression was diminished compared to wild type cells, indicating that NF-κB is required for full activation of BiP (Figure 2G). Using chromatin IP (ChIP), we found that p65 is able to bind to the promoter of BiP/GRP78 (Figure 2H), indicating that NF-κB activates transcription of BiP/GRP78 through direct interaction with BiP/GRP78 promoter. Furthermore, NF-κB binding to the promoter was IRE1 and PERK dependent demonstrating the importance of these components in activation of functional NF-κB during the UPR.

### The Kinase Activity of IRE1 Is Required for NF-κB Activation During ER Stress

**Figure 3:**
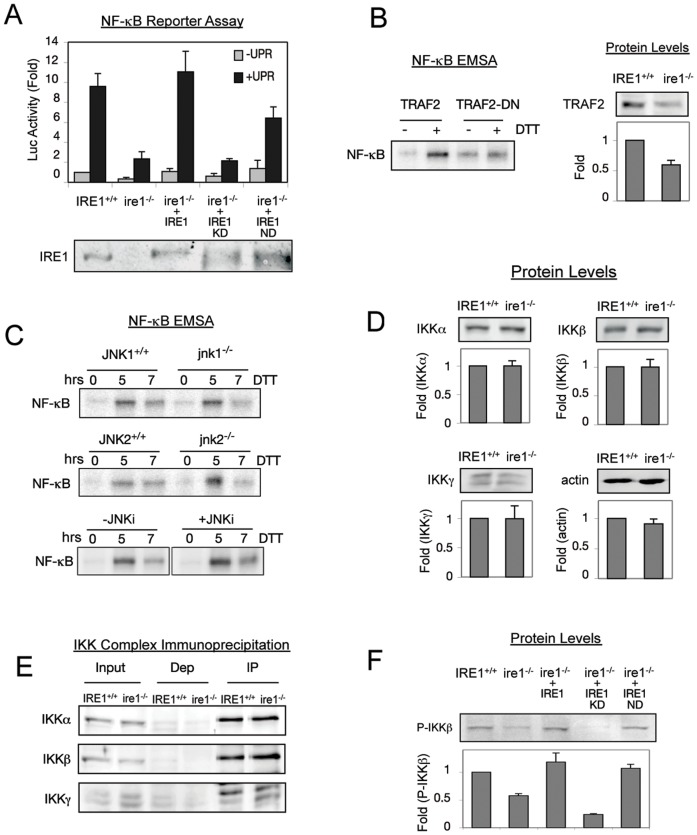
The kinase activity of IRE1 is required for NF-κB activation during the UPR. (A) Kinase dead IRE1 cannot rescue NF-κB activity in *ire1* ^−/−^ cells. *ire1* ^−/−^ cells were transfected with either WT IRE1, kinase dead IRE1 (IRE1-KD), or nuclease dead IRE1 (IRE1-ND). Western blots demonstrate successful transfection of IRE1 (bottom panel). IRE1^+/+^, *ire1* ^−/−^, and *ire1* ^−/−^transfected with different IRE1 mutants were treated with DTT and NF-κB luciferase reporter assays were performed. Fold change in luciferase activity in comparison to untreated *IRE1* ^+/+^ cells is shown. (B) NF-κB activation during the UPR requires TRAF2. WT cells were transfected with either WT TRAF2 (TRAF2) or dominant negative TRAF2 (TRAF2-DN), treated with DTT, and EMSA was used to determine the level of active NF-κB. Protein extracts from IRE1^+/+^ and *ire1* ^−/−^ cells were probed for TRAF2 by Western Blot. Fold change in TRAF2 levels in comparison to *IRE1* ^+/+^ is shown. (C) JNK is not involved in ER stress activation of NF-κB. WT, *jnk1^−/−^* or *jnk2^−/−^* cells were treated with DTT, and again EMSA was used to determine NF-κB activity. Furthermore, incubation of WT cells with 25 µM of SP600125 (JNKi), a well-established inhibitor of both JNK1/2 for up to 7 hrs did not affect activiation of NF-κB. (D) Protein levels of IKKα, IKKβ, and IKKγ in *ire1* ^−/−^ cells are equal to wild type levels. IKKα, IKKβ, and IKKγ protein levels in IRE1^+/+^ vs. *ire1* ^−/−^ cells were measured using Western Blot. Fold changes in protein levels in comparison to *IRE1* ^+/+^ are shown. (E) IRE1 does not affect the composition of the IKK complex. IKK complex was immunoprecipitated from IRE1^+/+^ and *ire1* ^−/−^ cells using anti-IKKγ antibody. The Input, depleted (Dep), and immunoprecipitated (IP) fractions are shown. Western blots were performed using antibodies against IKKα, IKKβ, and IKKγ. Efficient immunoprecipitation occurred as seen by low levels left behind in the depleted fraction. Equivalent amounts of each IKK subunit can be immunoprecipitated in WT and *ire1* ^−/−^ cells. (F) Basal phosphorylation of IKKβ is reduced in *ire1* ^−/−^ cells and cannot be rescued by kinase dead IRE1. *ire1* ^−/−^ cells were transfected with either WT IRE1, IRE1-KD, or IRE1-ND. P-IKKβ levels were determined by western blot in IRE1^+/+^, *ire1* ^−/−^, and transfected cells. Fold change in P-IKKβ in comparison to *IRE1* ^+/+^ is shown. For all experiments in this figure, quantitations are shown with standard error for at least three independent experiments.

Since cells lacking IRE1 showed severely decreased NF-κB during UPR, and IRE1 is a multi-functional protein, we wanted to examine the importance of IRE1 kinase and RNase activities. To separate each individual activity, we used a kinase dead form of IRE1 (IRE1-KD) and a nuclease dead IRE1 (IRE1-ND). IRE1-KD (K599A) was previously shown to inactivate IRE1 kinase both *in vivo* and *in vitro* [23]. Similarly, IRE1-ND (K907A), inactivated IRE1 RNase without affecting IRE1 kinase activity [24]. We transfected WT IRE1, kinase dead IRE1 (IRE1-KD), or nuclease dead IRE1 (IRE1-ND) into *ire1* ^−/−^ MEFs and determined NF-κB activation by NF-κB reporter luciferase assay. Western blotting shows that transfected IRE1 were expressed at similar levels as WT (Figure 3A, bottom panel). As shown previously (Figure 2F), *ire1* ^−/−^ MEFs displayed decreased NF-κB activation, however, transfecting wild type IRE1 rescues NF-κB activity (Figure 3A). Expression of IRE1-ND in *ire1* ^−/−^ MEFs essentially restored most of the NF-κB activity achieved by wild type IRE1 (Figure 3A). In contrast, expression of IRE1-KD was not able to rescue NF-κB activation in *ire1* ^−/−^ MEFs, demonstrating the importance of IRE1 kinase for NF-κB activation during the UPR.

While UPR activation results in IRE1 kinase activation, the functional contribution of IRE1's kinase in the UPR response has not been well characterized. However, the kinase function of IRE1 has been associated with its interaction with TRAF2, an adaptor protein that links IRE1 to c-JUN N-terminal kinase (JNK) activation [25]. Thus, we tested the involvement of TRAF2 and the JNK pathway in ER stress induced NF-κB activation. To test involvement of TRAF2, we used a dominant negative form of TRAF2 (TRAF2-DN) (Δ1–87), that has been shown to block IKK activation during canonical NF-κB pathway [26]. Transfection of TRAF2-DN into WT cells resulted in decreased levels of UPR-induced NF-κB activation (Figure 3B), indicating the importance of TRAF2 in the pathway. We also noticed that TRAF2 protein level itself was reduced in *ire1* ^−/−^ cells (Figure 3B). Thus, these results revealed that (i) TRAF2 is required for NF-κB activation and (ii) IRE1 may regulate cellular concentration of TRAF2. In contrast to TRAF2, however, loss of JNK in either *jnk1^−/−^* or *jnk2^−/−^* MEFs did not affect NF-κB activation significantly (Figure 3C). Furthermore, we inhibited both JNK1 and JNK2 using a well-established JNK Inhibitor (JNKi, SP600125) known to inhibit both forms of JNK [27], which has been previously shown to inhibit IRE1 dependent JNK activation during ER stress [28]. Treatment of cells with JNKi, which inhibited both JNK1 and JNK2 (Figure S4A), showed no effect on NF-κB activation (Figure 3C, +JNKi). Therefore, these data suggests that NF-κB activation during ER stress is mediated via IRE1-TRAF2 but not via JNK.

TRAF2 has been shown to regulate the activity of IKK in other signalling pathways [29]–[32]. We reasoned that diminished NF-κB activity in *ire1* ^−/−^ MEFs may come from altered compositions or states of IKK complex components, and thus, we determined the state of the IKK complex in WT versus *ire1* ^−/−^ cells. We first looked at protein levels of the IKK components to determine if IRE1 regulated their cellular concentrations. We found that protein levels of IKKα, IKKβ, and IKKγ were equivalent in WT and *ire1* ^−/−^ cells (Figure 3D), indicating that IRE1 does not affect protein levels of IKK components. Also, IRE1 may affect the composition of the IKK complex, so to test this, we immunoprecipitated the IKKγ and determined if equivalent amounts of IKKα and IKKβ were associated with it. We found that equal amounts of IKKα, IKKβ, and IKKγ were associated with each other in WT and *ire1* ^−/−^ cells (Figure 3E), indicating that IRE1 does not affect the physical composition of the IKK complex. Despite the presence of the intact IKK complex, however, IKKβ activity measured by its autophosphorylation was significantly diminished in *ire1^−/−^* cells (Figure 3F). Furthermore, the diminished phosphorylation can be rescued by WT and ND IRE1, but not KD IRE1, correllating with NF-κB activation (compare Figure 3A & 3F). Taken together, these results indicate that the kinase domain of IRE1 regulates NF-κB activation through TRAF2-IKK signalling.

### Basal IKK Kinase Activity is Diminished in the Absence of IRE1

**Figure 4:**
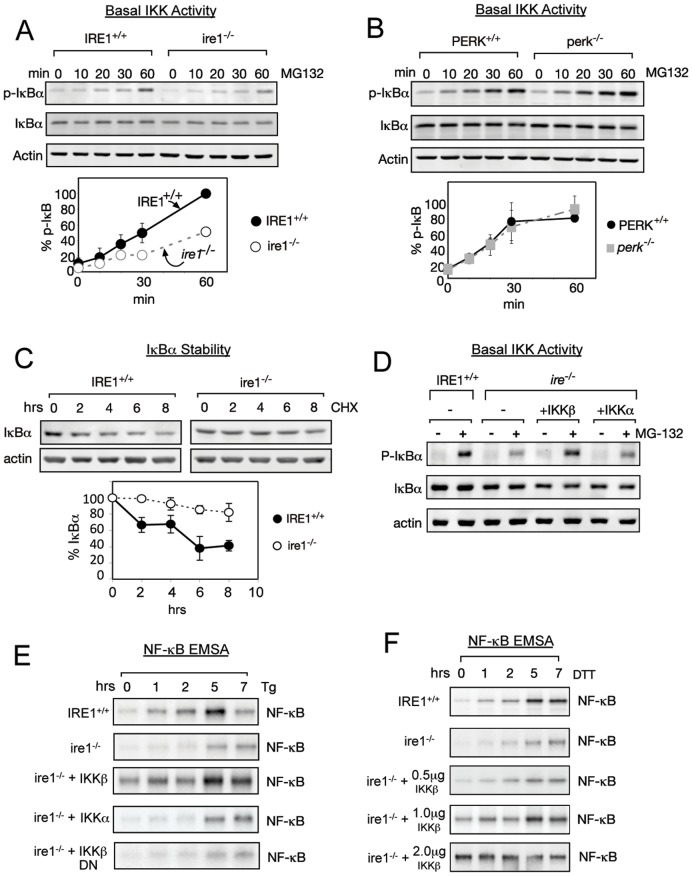
Basal IKK activity is decreased in cells lacking IRE1. (A) Cells lacking IRE1 have lower basal IKK activity. *IRE1* ^+/+^ (closed circle) and *ire1* ^−/−^ (open circle) MEFs were treated with MG132 for up to 60 min, and P-IκBα, total IκBα, and actin were measured by Western Blot. The rate of P- IκBα accumulation normalized to total IκBα was used as a measure of basal IKK activity. (B) PERK does not affect basal IKK activity. PERK^+/+^ (circle) and *perk* ^−/−^ (square) MEFs were treated with MG132 for up to 60 min, and P-IκBα, total IκBα, and actin were measured by Western Blot. The rate of P-IκBα accumulation normalized to total IκBα was used as a measure of basal IKK activity. (C) IκBα is more stable in *ire1* ^−/−^ cells. Translation was completely blocked in IRE1^+/+^ (closed circle) and *ire1* ^−/−^ (open circle) using 50 µg/ml cycloheximide (CHX). Decay of existing IκBα, in comparison to untreated conditions, was measured by western blot. For all experiments in this figure, quantitations are shown with standard error from at least three independent experiments. (D) Basal IKK activity can be rescued by expression of IKKβ. *ire1^−/−^* cells were transfected with IKKβ or IKKα. MG132 was added to either IRE1^+/+^, *ire1^−/−^*, or transfected cells for 60 min and accumulation of P-IκBα by western was used to measure basal IKK activity. Westerns for total IκBα and actin are shown. (E) NF-κB activation can be rescued by expression of IKKβ. *ire1^−/−^* cells were transfected with either IKKβ, IKKα, or dominant negative IKKβ (IKKβ DN). Either IRE1^+/+^, *ire1^−/−^*, or transfected cells were treated with Tg for up to 7 hrs. EMSA was used to determine levels of active NF-κB. (F) Modulating of basal IKK activity correspondingly activates NF-κB. *ire1^−/−^* cells were transfected with either 0.5 µg, 1 µg, or 2 µg of IKKβ. Either IRE1^+/+^, *ire1^−/−^*, or transfected cells were treated with DTT for up to 7 hrs and EMSA was used to determine NF-κB activity.

At the first glance, the lack of changes in either IKK complex itself or activity of IKK during ER stress seemed contradictory to involvement of IKK (Figure 1). Thus, we decided to further dissect the relationships between IKK, IRE1, and PERK. Although IKK is required for NF-κB activation, IKK activity did not increase during the UPR (compare Figure 1A/1B vs 1C/1D/1E). These observations suggested that the uninduced or *basal* IKK activity was sufficient to achieve NF-κB activation during the UPR. Recently, the importance of *basal* IKK activity in NF-κB activation by the ribotoxic stimulus UV was reported [21] [33], and thus, we tested the involvement of *basal* IKK kinase activity for UPR induced NF-κB. Since the traditional IKK kinase assay was not sensitive enough to detect changes in basal IKK activity (Figure 1E), we used accumulated P-IκBα levels as a readout for basal IKK activity. We measured *basal* IKK activity in *ire1^−/−^* and *perk* ^−/−^ cells, without any UPR inducing agents, to determine if loss of IRE1 or PERK affected *basal* IKK activity (Figure 4A&4B). We found that in *ire1^−/−^* MEFs, the rate of P-IκBα accumulation was 2-fold lower over the time course compared to WT (Figure 4A). In contrast, P-IκBα accumulation in *perk^−/−^* MEFs was identical to that of WT cells (Figure 4B), suggesting a role for IRE1, but not PERK, in retaining basal IKK activity.

Since IKK-mediated phosphorylation of IκBα leads to its degradation, these results predicted that IκBα in *ire1^−/−^* cells would have a longer half-life in uninduced resting or *basal* conditions. We assessed the half-life of IκBα in the presece of CHX, which prevented synthesis of new IκBα, and measured the rate of degradation of existing IκBα. Indeed, we found that IκBα disappeared more rapidly in WT than *ire1* ^−/−^ MEFs (Figure 4C), consistent with the idea that the half life of IκBα was longer in *ire1* ^−/−^ MEFs than WT cells. Together, these results suggested the exciting possibility that robust activation of NF-κB during the UPR requires a certain level of *basal* IKK activity, which needs to be present even prior to UPR induction. Furthermore, these results also argued that IRE1 affected basal IKK activity.

### Restoring Basal IKK Activity Can Rescue NF-κB Activation in *ire1* ^−/−^ Cells

The relationship between IRE1 and IKK suggests that decreased NF-κB activation in *ire1* ^−/−^ MEFs is caused by decreased basal IKK activity. To further test this idea, we increased basal IKK activity in *ire1* ^−/−^ MEFs by increasing the amount of IKK and determined if efficient NF-κB activation was restored. To this end, *ire1^−/−^* cells were transfected with extra copies of either IKKα or IKKβ. Indeed, we found that expression of IKKß, but not IKKα, in *ire1^−/−^* MEFs restored the basal level of IKK activity as measured by accumulation of P-IκBα in the presence of MG132 (Figure 4D). Consequently, IKKβ, but not IKKα, was able to rescue NF-κB activation to wild type levels in the *ire1* ^−/−^ cells (Figure 4E). Since IKKβ, but not IKKα, is the main kinase phosphorylating IκBα in cannonical NF-κB activation, this result suggests that the UPR utilzes cannonical IKK activity for NF-κB activation. In addition, the kinase activity of IKK is needed for activation of NF-κB by UPR, because transfection of a kinase-dead mutant of IKKβ did not rescue NF-κB activity to wild type levels in *ire1^−/−^* MEFs (Figure 4E, +IKKβ DN). Overall, these results highlight the importance of IKKβ kinase function in mediating UPR activated NF-κB.

These experiments reveal that *basal* IKK activity stimulates NF-κB activation during the UPR. To demonstrate the functional importance of regulating *basal* IKK activity, we altered levels of basal IKK activity by transfecting of increasing amounts of IKKβ into *ire1^−/−^* MEFs and inducing the UPR (Figure 4F). Transfecting 1 µg of IKKβ plasmid resulted in NF-κB activation similar to WT IRE1^+/+^ cells. Curiously, at high levels of IKK activity, while active NF-κB level was elevated at the basal level prior to UPR, activation of the UPR did not result in further increased NF-κB activity (Figure 4F, 2 µg, compare 0 hr vs 1, 2, 5 & 7 hrs). Levels of transfected IKKβ were confirmed by western blot (Figure S4B). These results highlight the importance of considering basal IKK activity in mediating UPR induced NF-κB activation. Taken together, our results show that basal IKK activity, maintained by IRE1, is critical to activate NF-κB when PERK-induced translation inhibition occurs. Furthermore, this finding has implications in systems which alter IKK basal activity such as drug development of IKK inhibitors.

### ER Stress Can Override Inhibition of an IKK Inhibiting Drug

**Figure 5:**
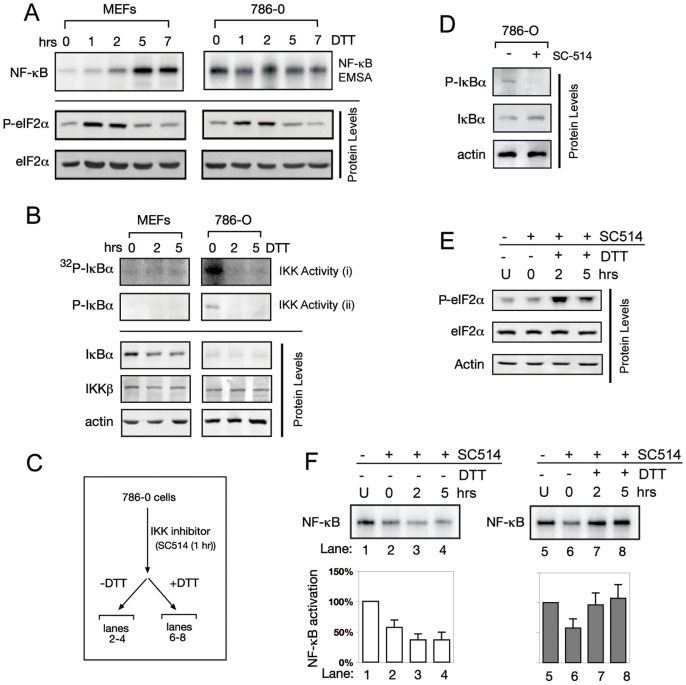
Activation of the UPR can override the inhibition of NF-κB by IKK inhibitors in cancer cells with elevated IKK activity. (A) 786-0 cells have constitutively active NF-κB. WT MEFs or 786-O cells were induced with DTT for up to 7 hrs. NF-κB activity was measured by EMSA. Also, levels of P-eIF2α or total eIF2α were measured by western blot. MEFs and 786-0 cells show similar patterns of eIF2α phosphorylation indicating an intact UPR in 786-0 cells. (B) 786-0 cells have increased basal IKK activity. IKK activity was measured two ways in WT MEFs or 786-0 cells. IKK activity (i) was measured by incubating IKK complex IP'd from cells with recombinant IκBα and γ^32^P-ATP, whereas IKK activity (ii) shows P-IκBα present in cell extracts measured by western blot. Also, protein levels of P-IκBα, total IκBα, IKKß, and actin were determined by Western Blot. (C) Schematic for following experiments. 786-0 cells were treated with an IKK inhibitor, SC514 for 1 h to reduce IKK activity, then cells were treated without or with DTT for up to 5 hours. (D) SC514 decreases IKK activity. 786-O cells were treated with SC514 for 1 hour, and westerns against P-IκBα, total IκBα, and actin were performed. A reduction in P-IκBα and increase in total IκBα were seen corresponding to a decrease in IKK activity. (E) SC514 does not affect UPR signalling. 786-0 cells were incubated with SC514 for 1 h (‘0’), then DTT was added up to 5 hrs. Westerns against P-eIF2α, total eIF2α, and actin were done. P-eIF2α levels did not change upon addition of SC514, but increased with DTT, indicating SC514 does not alter the normal UPR response. ‘U’ indicates untreated conditions. (F) UPR signalling overrides inhibition by SC514. 786-0 cells were incubated with SC514 for 1 hr (‘0’: lanes 2 and 6). Cells were then incubated either without DTT (lanes 3–4) or with DTT (lanes 7–8) still in the presence of SC514. EMSA was performed to measure NF-κB activity. SC514 resulted in a decrease of NF-κB activity (lanes 2–4), but induction of UPR caused activation of NF-κB even in presence of SC514 (lanes 6–8). ‘U’ indicates untreated conditions. Quantitation with standard error is shown using NF-κB activity in untreated 786-0 cells as 100% and represents at least three independent experiments.

Certain cancer cells have been reported to display elevated NF-κB/IKK activity [34], and drugs have been developed that which aim to decrease IKK activity back to basal levels [35]. However, our results suggest that basal IKK activity is sufficient to activate NF-κB during the UPR, so we wanted to test if UPR activation can counteract the effect of the IKK inhibiting drug. 786-0 renal carcinoma cells were previously characterized to have constitutively active NF-κB activity [36], [37]. EMSA assays revealed that basal NF-κB activity was significantly elevated in 786-0 cells as compared to MEFs (Figure 5A, 0 h). UPR induction in 786-0 cells was unable to further activate NF-κB (Figure 5A), similar to *ire1^−/−^* MEFs transfected with high levels of IKKβ (Figure 4F, 2 µg) providing additional support for the significance of uninduced IKK activity in determining the outcome of NF-κB signaling. Also, 786-0 cells were able to activate PERK normally during the UPR as measured by P-eIF2α (Figure 5A), indicating a normal UPR response. Uninduced 786-0 cells also show elevated IKK activity as measured by IKK kinase assay, increased P-IκBα, and decreased total IκBα levels (Figure 5B), making these cells a good candidate for targeting with IKK inhibitors.

Our current study predicts that, in 786-0 cells, NF-κB becomes activatable by UPR if elevated basal IKK activity is reduced to its normal *basal* level by an IKK inhibitor. Since UPR becomes induced by the tumor microenvironment, using an IKK inhibitor as treatment of such cancer cells may not be an effective cancer treatment. Instead, the reduction in elevated IKK activity by the inhibitor would allow cells to activate NF-κB in response to the UPR inducing microenvrionment of the tumor, rendering the inhibitor ineffective. To test our hypothesis, we treated 786-0 cells for 1 hr with a well-characterized IKK inhibitor, SC-514 [38] (Figure 5C, schematic), and found that SC-514 treatment diminished both basal IKK (Figure 5D) and NF-κB activities and continued to suppress NF-κB throughout the time course (Figure 5F, lanes 1–4). In contrast, induction of UPR by DTT after SC-514 treatment resulted in robust activation of NF-κB (Figure 5F, compare lanes 2–4 for no UPR, vs lanes 6–8 with DTT), even in the continued presence of the IKK inhibitor. Note that SC-514 did not alter activity of PERK, judging from p-eIF2α (Figure 5E vs 5A), and thus, differences in these effects were not due to disruptions in translational regulation. These results are consistent with our hypothesis that the decrease in elevated IKK/NF-κB to basal levels in cancer cells by an IKK inhibitor allowed them to respond to UPR by activating NF-κB. Taken together, these results provide additional support for the idea that *basal* IKK activity is a critical factor in UPR induced NF-κB activation.

## Discussion

**Figure 6:**
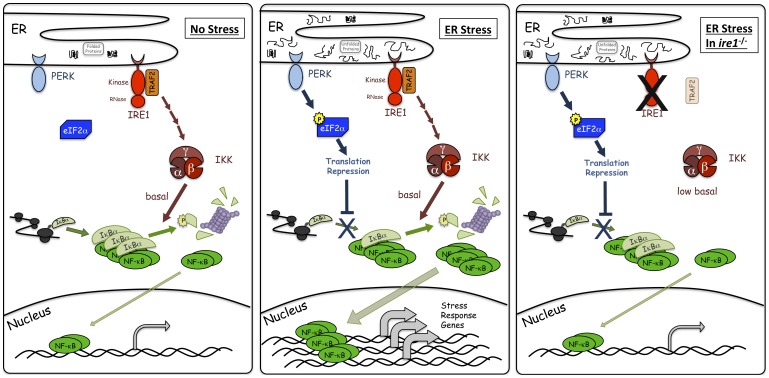
Model for NF-κB activation during ER stress. Under unstressed conditions, IκBα is being synthesized, binds, and inhibits NF-κB. IRE1, through TRAF2, is maintaining basal IKK activity which is responsible for phosphorylating a subset of IκBα leading to proteosomal degradation and basal NF-κB activity. However, most of the NF-κB is sequestered by IκBα. During ER stress, PERK phosphorylation of eIF2α leads to translation repression which then prevents synthesis of new IκBα and contributes to a decrease in IκBα levels and corresponding increase in free NF-κB levels. Additionally, basal IKK activity is responsible for phosphorylating and degrading the existing IκBα, including IκBα bound to NF-κB, causing a more dramatic decrease in IκBα levels resulting in an even greater amount of free NF-κB. Free NF-κB can then translocate to the nucleus to assist in transcriptional activation of stress response genes. During ER stress in cells with decreased basal IKK, such as *ire1* ^−/−^ cells, basal IKK is considerably reduced. PERK mediated translation inhibition alone is unable to reduce IκBα levels enough to allow for a significant amount of free NF-κB. Thus, combined inputs from both PERK are IRE1 are required for full activation of NF-κB during ER stress. It should be noted that the possibility remains that additional element(s) beyond both PERK induced translation repression and IRE1 regulation of basal IKK/IκBα stability, may also contribute to overall activation of NF-κB during ER stress.

The NF-κB transcription factor family regulates genes involved in a wide array of processes including inflammation, immune responses, apoptosis, and turmorigenesis [17]. NF-κB is also activated by certain types of ER stress [14] [13] [12]. These previous studies have reported involvement of either PERK or IRE1 in UPR-induced NF-κB activation. However, studies examining the role of PERK had never investigated significance of IRE1, and conversely, studies reported the role of IRE1 did not examine its relationships to involvement of PERK. Furthermore, those studies were performed in different cell types. Thus the relationships between PERK and IRE1 for NF-κB activation in response to ER stress remained unclear. Here, we have shown that optimal activation of NF-κB during ER stress requires inputs from both IRE1 and PERK activities (Figure 6). In agreement with previous studies, we found that global translation repression induced by PERK-dependent phosphorylation of eIF2α plays a critical role in activation of NF-κB during ER stress (Figure 2A&2B). Furthermore, we have shown that the extent of transltional inhibition propotionally correllates with the level of NF-κB activation (Figure S3A). One of the key findings of our study is that IRE1-dependent homeostatic regulation of basal IKK activity is necessary for effective activation of NF-κB by PERK. An increase or decrease in basal IKK activity affects the ability of the PERK signaling branch to effectively activate NF-κB when ER stress is imposed. Although we do not detect an increase in IKK activity during the UPR in MEFs, a previous study reported that UPR-activated IRE1 caused activation of IKK in MCF-7 cells, a breast cancer cell line [18], raising the possibility that oncogenic transformation may intensify the IRE1 contribution to IKK activity. Furthermore, our subsequent analyses showing the ability of both a nuclease dead IRE1 and IKKβ to restore NF-κB activation in *ire1^−/−^* knockout cells in response to ER stress (Figures 3A and 4D–4F) have provided additional support for the involvement of IRE1 in basal IKK regulation. The ability of IRE1 to modulate *basal* IKK activity suggests that IRE1 is never completely inactive or “OFF”, even in the absence of overt ER stress, and that IRE1 has an intrinsic housekeeping function. Previously, we reported that a basal state IRE1 function is also required for efficient cytokinesis during mitosis in *S. cerevisiae* [39]. There, we proposed that IRE1 activity is modulated in a “dimmer switch” fashion, rather than an “ON/OFF switch”. Furthermore, we have reported the role of basal activity in the NF-κB pathway during the ribotoxic stimulus UV [21]. Taken all together, therefore, we propose that the optimal activation of NF-κB during the UPR is mediated by inputs from both PERK and IRE1, and by altering either one or both of these inputs, the extent of NF-κB activation can be fine-tuned, allowing for a dynamic response to ER stress.

One of the questions that remains to be answered is the molecular mechanism by which IRE1 regulates basal IKK activity. Previously, UPR activated IRE1 has been shown to associate with TRAF2 and JNK through its kinase, but not RNase, domain [25]. In our current study, we found that the cellular TRAF2 level was significantly diminished in *ire1^−/−^* knockout cells (Figures 3B). Similarly, NF-κB activation upon UPR induction was also diminished in the absence of IRE1 (Figure 2D). We have also shown that the IRE1 kinase, but not RNase, is required for basal IKKβ phosphorylation (Figure 3F) and NF-κB activation (Figure 3A). Curiously, however, JNK plays little role in UPR-induced NF-κB activation (Figure 3C). Taken together, these data are consistent with TRAF2 involvement in IRE1 activation of IKK and NF-κB, pointing to the presence of a physical interactions between IRE1, IKK and TRAF2. While previous studies have reported a physical interaction between IRE1, TRAF2, and IKK only takes place once UPR is activated [18], results described here suggest that these interactions, if they occur, should be present at the basal level. Thus, this complex may exist transiently or be present at low levels during basal conditions. In fact, our attempts to detect physical interactions between IRE1 and either IKKβ or the IKK complex at the basal state by co-immunoprecipitation with IRE1 have failed even in the presence of reversible crosslinker (data not shown), and more sensitive experimental methods may require for detection of such interaction.

eIF2α can be phosphorylated by multiple kinases activated by various stresses. For example, during viral infection, double-stranded RNA can induce eIF2α phosphorylation by PKR while generation of un-charged tRNAs during nutrient deprivation causes eIF2α phosphorylation by CGN2 [40]. Furthermore, celluar stresses such as UV damage and hypoxia may activate multiple eIF2α kinases [41]–[42]. A common feature between these stress responses is that they all contain a translational inhibition component and have all been shown to activate NF-κB [43]. Our study here suggests that activation of NF-κB in such cases that repress global translation including viral infection or nutrient deprivation also requires the contribution of basal IKK activity. In these cases, an additional component(s) other than IRE1 may exist to regulate basal IKK activity. Furthermore, basal IKK activity may also be regulated via TRAF2, similarly to IRE1.

Recently, links between UPR and cancer development are becoming increasingly clear. For example, overexpression of spliced XBP1 is tightly correlated with multiple myelomas [44]. Furthermore, cells lacking PERK or XBP1 form significantly smaller tumors in nude mice [5] [4]. Also, a variety of microenvironments conducive to tumor development including hypoxia, nutrient starvation, and acidosis, all have been shown to induce UPR [45], [46], [47], [48]. Together, these demonstrate that the UPR is a critical component of tumor development. As NF-κB also promotes tumor survival, NF-κB inhibitors are often used to suppress elevated NF-κB in cancer cells, in order to make such tumor more susceptible to chemotherapies [49]. However, our studies suggest that administration of NF-κB inhibitors could lender tumors more “responsive” to UPR and thus, to UPR inducing microenvironments specifically through PERK. Our results suggest that PERK inhibitors in combination with IKK inhibitors would provide rather more effective NF-κB suppression.

## Materials and Methods

### Cell culture and treatment

IKKα/β^+/+^ and *ikk*α/β*^−/−^* [19], *ikk*α*^−/−^* [50], *ikk*β*^−/−^* [21], IκBα WT and IκBαSR [21], IRE1*^+/+^ and ire1^−/−^* [51], PERK^+/+^ and *perk^−/−^* [52], *p65* ^−/−^ [53], JNK^+/+^, *jnk1* ^−/−^ and *jnk2* ^−/−^ [54] MEFs were cultured in DMEM media (Cellgro). 786-O [55] cells were cultured in RPMI 1640 (Cellgro). All media was supplemented with 10% fetal calf serum (Gibco), 100 U/ml penicillin, and 100 µg/ml streptomycin (Mediatech). Cells were grown in 5% CO_2_ at 37°C. Cells were treated with 200 nM thapsigargin (Calbiochem), 1 mM DTT (Fisher), 50 µg/ml cycloheximide (Sigma), 25 µM MG-132 (Calbiochem), 100 µM SC-514 (Calbiochem), or 20 ng/ml tumor necrosis factor α (TNFα) (Sigma) for the indicated amount of time. JNK inhibitor SP600125 (Cayman Chemical) was used at concentration of 25 µM.

### EMSA

NF-κB activity was measured by an electrophoretic mobility shift assay (EMSA) as previously described [56]. Nuclear protein was incubated at room temperature for 15 min with 0.01 pmol of ^32^P labeled probe containing an NF-κB binding site (AGTTGAGG**GGACTTTCC**CAGGC) in binding buffer (10 mM Tris (pH 7.5), 50 mM NaCl, 10% glycerol, 1% NP-40, 1 mM EDTA, 0.1 µg/µl poly-dI**^.^**dC) as described detail in Pahl and Baeuerle, 1995. Complexes were separated by gel electrophoresis using a 5% non-denaturing polyacrylamide gel and visualized by autoradiography. Competition assays were performed using a single stranded probe that forms a double stranded hairpin structure containing an NF-κB binding site. The sequences of the wild type and mutant competitors are WT:(CTG**GGGACTTTCC**AGGTTAGCTTCCT**GGAAAGTCCC**CAG) and the mutant:(CTG***TCT*** **ACTTTCC**AGGTTAGCTTCCT**GGAAAGT** ***AGA***CAG). Supershift assays were performed using antibodies against p65 and p50 of NF-κB (Santa Cruz Biotech) added to the binding mixture and incubated on ice for 1 hr at 4°C, and separated by electrophoresis using a 5% non-denaturing polyacrylamide gel and visualized by autoradiography. As a control, an Oct-1 probe (TGTCGA**ATGCAAAT**CACTAGAA) was used on the same nuclear extracts to determine that total protein in the extract was similar.

### Western blot

After treatment, cells were washed with ice cold PBS twice and the lysed using RIPA buffer (20 mM HEPES (pH 7.4), 150 mM NaCl, 1 mM EDTA, 1% NP-40, 0.25% NaDeoxycholate, 0.1% SDS, 10 mM NaF, 1 mM NaVO4, 1 mM PMSF, 1 mM PMSF, 100 u/ml Aprotinin, 1.4 µg/ml Pepstatin, 1 µg/ml Leupeptin). The protein concentration was then normalized by BCA Assay (Pierce). Samples were analyzed by SDS-PAGE and transferred to nitrocellulose and probed with antibodies against NF-κB p65 subunit (Santa Cruz), p50 (Santa Cruz), IκBα (Santa Cruz), phospho-IκBα (Cell Signaling), eIF2α (Cell Signaling), phospho-eIF2α (Stressgen), IKKα (Santa Cruz), IKKβ (Biosource), IKKγ (Santa Cruz), P-IKK (Cell Signalling), TRAF2, (Santa Cruz), P-IKKβ (Cell Signalling), P-JNK (Santa Cruz), and actin (Sigma).

### ^35^S Labeling

After treatment, cell were labeled with 50 µCi/ml ^35^S (Trans ^35^S-Label, MP Biomedicals) for 10 min, and washed twice with cold PBS containing non radio-labeled methionine. 15 µg of total protein was separated by SDS-PAGE and visualized by staining with coomassie blue staining (CB). Cellular translation levels were measured by incorporation of radiolabeled amino acids using autoradiography.

### Transfections

Cells were plated to 50% confluency in 10 cm plates the day before transfection. Plasmids were transfected using the Effectene (Qiagen) system according to manufacturer's instructions for 48 hrs.

### Dual Luciferase Assays

Cells were transfected with 1 µg of a plasmid containing 3XκB-Luc reporter. As an internal control, cells were also co-transfected with 0.05 µg of a plasmid with a *Renilla* luciferase gene driven by an SV40 promoter. Transfections were done in triplicate, and after 48 h cells were treated as indicated. Luciferase assays were carried out using the Dual-Luciferase Reporter Assay (Promega) according to manufacturer's instructions. Luciferase activity was measured by luminomiter (Analytical Luminescence Laboratory, Monolight, model 2010). Values of samples were normalized to *Renilla* luciferase. Values shown are averages and standard error from at least three independent experiments.

### IKK Kinase Assay

IKK activity was measured by IKK kinase assays as previously described [56]. After treatment, cells were washed twice with cold PBS, and cytoplasmic extracts were taken using Cytolplasmic Extract Buffer (10 mM HEPES-KOH pH 7.9, 250 mM NaCl, 1 mM EDTA, 0.5% NP-40, 0.2% Tween 20, 2 mM DTT, 1 mM PMSF, 20 mM β-glycerophosphate, 10 mM NaF, 0.1 mM Na_3_VO_4_). IKK complexes were then immunoprecipitated using an antibody against IKKγ (Pharmingen). Cytoplasmic extracts before and after immunoprecipitation were run on a western blot and probed for IKKα (Santa Cruz) to check for immunoprecipitation efficiency. IKK complexes were then incubated with 10uCi γ^32^P-ATP, recombinant IκBα in Kinase Buffer (20 mM HEPES pH 7.7, 20 mM β-glycerophosphate, 100 mM NaCl, 100 µM Na_3_VO_4_, 10 mM MgCl_2_, 10 mM NaF, 1 mM PMSF, 2 mM DTT, 20 µM cold ATP) at 30°C for 30 min. Samples were then run on SDS-PAGE gels and phosphorylated IκBα was visualized by autoradiography.

### RNA extraction, RT, and quantitative PCR

Total RNA was prepared using RNeasy Mini Kit (Qiagen) and treated with DNase (Qiagen) according to manufacturer's instructions. One microgram of total RNA was then reverse transcribed using ThermoScript reverse transcriptase (Invitrogen) according to manufacturer's instructions to obtain cDNA. For quantitative PCR, 5 ng of input cDNA was analyzed in triplicate per sample for each primer pair. All reactions were performed using SYBR Green PCR Master Mix (Applied Biosystems) and 400 nM of each primer per reaction in a total volume of 25 µl. The reaction was performed using default cycling parameters on an ABI Prism 7200 Sequence Detector. A standard curve composed of five-fold serial dilutions of concentrated cDNA was included in each qPCR run for each primer. *GRP78/BiP primer*: F-CCATCCCGTGGCATAAACC, R-GGAATCAGTTTGGTCATGACACC. Since the SYBR Green system is used, it is important to have a single product being amplified so a melting curve analysis was performed after each run to confirm amplification of a single product. Expression of each gene was normalized to 18S and expressed as fold induction. Values are mean±s.e.m of at least three independent experiments.

### Chromatin Immunoprecipitation

ChIP was performed essentially as described [57]. Briefly, wild type, *ire1* ^−/−^, *perk* ^−/−^, and *nf-*κB^−/−^ cells were treated with 200 nM Thapsigargin and fixed with 1% formaldehyde (Sigma). Cells were then sonicated to obtain approximately 500 bp fragments, and lysate was cleared of debris by centrifugation. Sample was then precleared with protein-G (Upstate) beads blocked with sheared salmon sperm DNA (New for 2 h twice. Immunoprecipitation was done overnight at 4°C with 1 µg p65 antibody (Santa Cruz Biotech). For the negative control, a mouse IgG (Bio-Rad) was used instead p65 antibody. Complexes were recovered by protein-G beads blocked with salmon sperm DNA, washed under stringent conditions, then decrosslinked at 65°C overnight. While decrosslinking, samples were also eluted with 1% SDS. Samples were then treated with Proteinase K at 55°C for 2 hrs. DNA was extracted by phenol/chloroform and PCR was performed. Primers were designed targeting the promoter regions of genes and contain at least one putative NF-κB binding site. *BiP/GRP78 primer* F- GGCGTAGCAATGACGTGAG, R- GCCACTCGCCTTATATACCC.

## Supporting Information

Figure S1 **ER stress activated NF-κB contains p65/p50.** (A) DTT activates NF-κB. Wild type MEFs were treated with DTT for up to 7 hrs and NF-κB activation was measured by EMSA (left panel). Right panel: For the Competition Assay, the 5 hr DTT sample was incubated with a non-radiolabelled oligo containing a wild type NF-κB binding site (κB wt). The correct NF-κB band is then competed out by the cold probe resulting in a loss of the signal. The band labeled (*) cannot be competed out by the cold probe containing a wild type NF-κB binding site, hence it is designated as a background band. Its identity as a background band is further confirmed by supershift assays as described below. When a cold competitor containing a mutation in critical residues of the κB binding sites are added (κB mt), this does not compete out the NF-κB band indicating that NF-κB is binding to the radiolabelled wild type probe. (B) EMSA and Competition Assay as described in (A) for cells treated with Thapsigarin (Tg) instead of DTT. Results are similar to the DTT treated cells as described above. (C) Supershift assays identifying the active form of NF-κB contains p65 and p50. Eihter the 5 hr DTT or TG (thapsigargin) samples were incubated with antibodies against p65, p50 or both p65 and p50 for 1 hr. Samples were then run on a gel. Incuabation of p65 resulted in a loss of the NF-κB band, and the appearance of a higher band corresponding to the probe/p65/antibody complex which has a slower mobility on the gel (shifted p65). Incubation with p50 also resulted in disappearance of the NF-κB band and the appearance of a shifted band (shifted p50). Incubation of both p65 and p50 antibodies resulted in disappearance of the NF-κB band and appearance of two shifted bands. This indicates that the activated NF-κB contains p65 and p50. The band labeled (*) could not be shifted by either p65 or p50. (D) Nuclear localization of NF-κB during TG and TNFα treatment. Cells were treated with TG for 1 hr or TNFα for 15 min. Immunofluorescence was then performed with antibodies against p65 and DAPI to detect nuclei. Untreated cells show cytosolic localization of p65, but treatment of both TG or TNFα resulted in nulcear localization of p65. (E) IKKß kinase activity is required for ER stress induced NF-κB activation. (Top) IKKß, but not IKKα, is required for NF-κB activity. WT, *ikk*α*^−/−^*, or *ikk*ß^−/−^ cells were treated with Tg for up to 5 hrs. Nuclear extracts were collected and EMSA was performed using radiolabelled DNA containing an NF-κB binding site. *ikk*α*^−/−^* cells induced NF-κB similar to WT cells, however, *ikk*β^−/−^ cells were unable to induce NF-κB, demonstrating that IKKβ, but not IKKα is required for NF-κB activation during ER stress. (Bottom) IKKβ kinase activity is required for NF-κB activation during ER stress. *ikk*β*^−/−^* cells were transfected with either WT IKKβ or kinase dead IKKβ (IKKβ DN) and treated with Tg for 2 hrs. *ikk*β*^−/−^* and *ikk*β*^−/−^* transfected with kinase dead IKKβ are unable to induce NF-κB. However, *ikk*β*^−/−^* cells transfected with WT IKKβ are able to induce NF-κB during ER stress, indicating that IKKβ kinase activity is required for NF-κB activation during ER stress.
(TIF)

Figure S2 **NF-κB activation in multiple cell types: NIH 3T3 and CHO.** (A) NF-κB is activated by ER stress in NIH 3T3 cells. NIH 3T3 cells were treated with either DTT or TG for up to 5 hrs, nuclear extracts were prepared, and EMSAs were performed to measure NF-κB activation. NF-κB was activated in treatments with both DTT and TG. (*) and (**) denote background bands as determined by competition assays. (B) Competition assay for NIH 3T3 cells. The 2 hr sample was taken and incubated with a cold oligo containing a wild type or mutant NF-κB binding site. The p65/p50 can be competed out by the wild type competitor but not the mutant competitor demonstrating that the complex observed was p65/p50. Although the band labeled (*) can be competed out by the wild type cold competitor, it can also be competed out by the mutant competitor signifying that it is unlikely to be NF-κB. The band labeled (**) cannot be competed out by the wild type competitor demonstrating that it is a background band. (C) CHO cells were treated with either DTT, TG, or TNFα for up to 5 hrs, nuclear extracts were prepared and EMSAs were performed to measure NF-κB activation. The main form of NF-κB activated by DTT was p50/p50 while TG activated the p65/p50 form of NF-κB as determined by competition and supershift assays in (D). (D) Competition and supershift assays of NF-κB activated in CHO cells. The 2 hr sample was used and incubated with a wild type or mutant cold competitor. Two bands (p65/p50 and p50/p50) were able to be competed by the cold wild type competitor, but were not able to be competed by the cold mutant competitor, indicating that these bands are NF-κB bands. Also, supershift assays reveal the presence of two forms of NF-κB, p65/p50 and p50/p50 as indicated. The p65/p50 band can be shifted by antibodies against both p65 and p50. The p50/p50 band does not shift when only p65 antibody is added, but can be shifted by the p50 antibody, indicating the composistion of the band contains p50 but not p65. (E) Total IκBα levels decrease but P-IκBα does not increase during the UPR in CHO cells. CHO cells were treated with DTT or Tg for up to 5 hrs and protein was extracted. Western blots were performed against P-IκBα, total IκBα, and actin. P-IκBα levels do not increase, similar to results using MEFs. Also, IκBα levels decrease during both DTT and Tg treatment similar to MEFs. Actin was used as a loading control.
(TIF)

Figure S3 **Translation inhibition determines NF-κB activation and addition of IRE1 can rescue NF-κB activation and XBP1 splicing in ire1^−/−^ cells.** (A) Degree of translation inhibition determines the strength of NF-κB activation. Increasing concentrations of CHX were used to inhibit translation at different levels. We found that 0.01 µg/ml CHX will inhibit 40% of global translation, 0.1 µg/ml CHX inhibits 75% of global translation, and 50 µg/ml inhibits 100% of global translation using ^35^S labelling experiments (data not shown). Wild type MEFs were then treated with these concentrations of CHX, and NF-κB activation was determined by EMSA. Increasing the degree of global translation inhibition results in increasing NF-κB activity. 40% translation inhibtion does not increase NF-κB activation significantly. 75% translation inhibition shows a moderate activation of NF-κB. 100% translation inhibition shows the strongest NF-κB activation. (B) Adding back IRE1 can rescue NF-κB activation in *ire1* ^−/−^ cells. *ire1* ^−/−^ cells were transfected with increasing amounts of IRE1 plasmid up to 2 µg for 48 hrs. Cells were then treated with DTT for 5 hours, nuclear extracts were prepared, and EMSA assays were performed to determine NF-κB activation in IRE1^+/+^ or transfected cells during DTT treatment. Transfecting increasing amounts of IRE1 led to increasing activation of NF-κB. Quantitations are shown, and at least three independent experiments were performed. (C) Adding back IRE1 can rescue XBP1 splicing in *ire1* ^−/−^ cells. RNA was collected from samples in (B) and RT-PCR was performed to determine XBP1 splicing. Unspliced (u) XBP1 contains a 26 nt intron which results in a higher band as compared to the spliced (s) XBP1. *ire1* ^−/−^ cells are unable to splice XBP1 while WT cells splice XBP1 efficiently during DTT treatment. Adding back IRE1 resulted in increasing rescue of XBP1 splicing in *ire1* ^−/−^ cells corresponding to increasing rescue of NF-κB activation.
(TIF)

Figure S4 **JNK Inhibition and Transfecting IKKβ increases basal IKK activity.** (A) JNK inhibitor (JNKi, SP600125) inhibits JNK activation during ER stress. JNKi was added to cells as in Figure 3C. Protein extracts were taken and western blots were performed using antibody detecting active, phosphorylated JNK. Cells treated with DTT showed activation of both JNK1 and JNK2, but when JNKi was added, both JNK1 and JNK2 activation was inhibited. (B) In Figure 4F, transfecting increasing amounts of IKKβ led to increasing basal IKK activity. In order to determine how much IKKβ was transfected, western blots were performed to derermine if equivalent amounts of IKKβ were expressed. Transfecting 0.5 µg plasmid expressed an amount similar to endogenous IKKβ, while 1 and 2 µg expressed significantly more IKKβ.
(TIF)
