# Supplementary figures and images for: ER Stress Activates NF-κB by Integrating Functions of Basal IKK Activity, IRE1 and PERK
Source: PLoS One. 2012 Oct 26;7(10):e45078. doi: 10.1371/journal.pone.0045078 (PMC3482226; doi:10.1371/journal.pone.0045078)

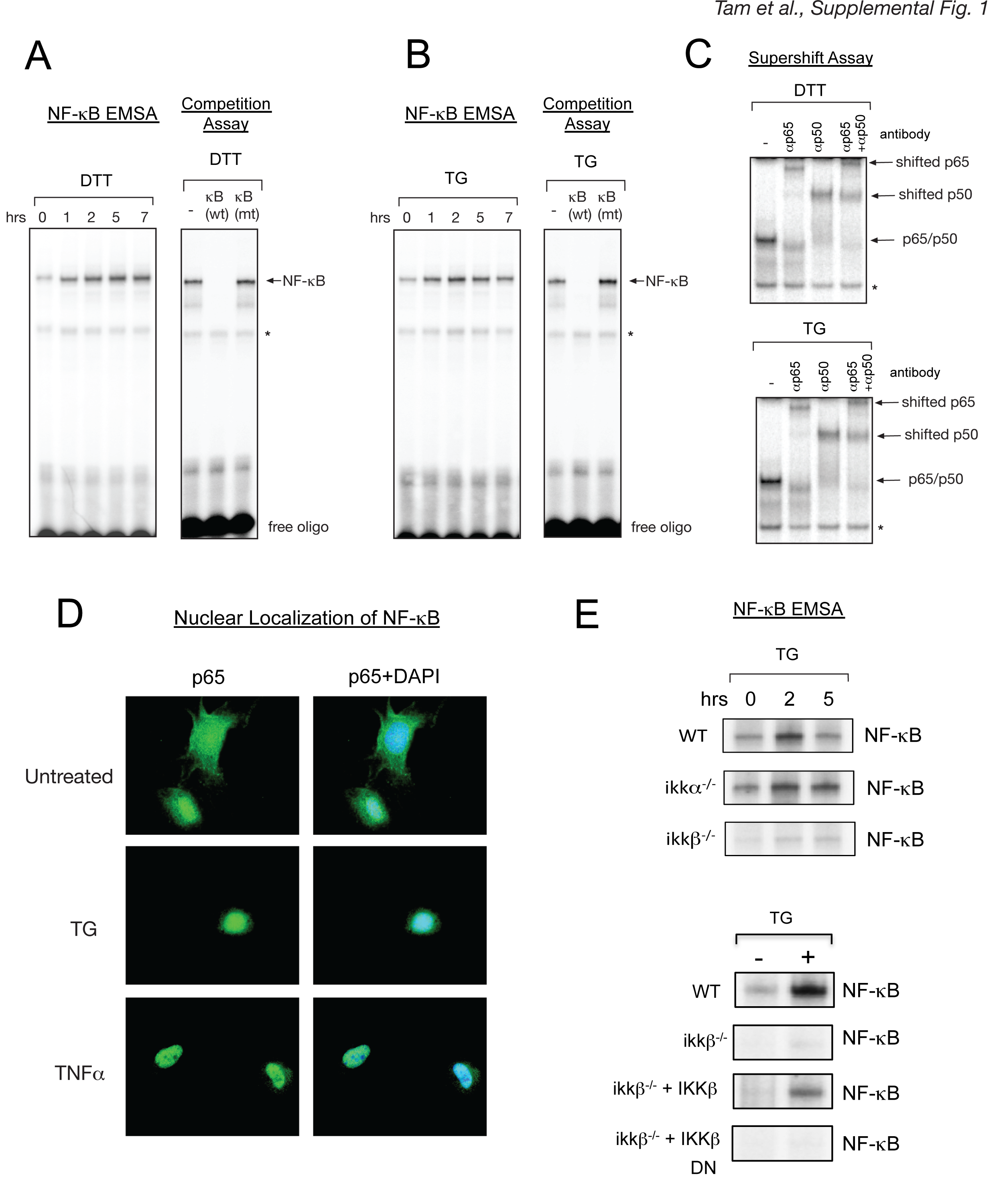

Supplement: Figure S1 — ER stress activated NF-κB contains p65/p50. (A) DTT activates NF-κB. Wild type MEFs were treated with DTT for up to 7 hrs and NF-κB activation was measured by EMSA (left panel). Right panel: For the Competition Assay, the 5 hr DTT sample was incubated with a non-radiolabelled oligo containing a wild type NF-κB binding site (κB wt). The correct NF-κB band is then competed out by the cold probe resulting in a loss of the signal. The band labeled (*) cannot be competed out by the cold probe containing a wild type NF-κB binding site, hence it is designated as a background band. Its identity as a background band is further confirmed by supershift assays as described below. When a cold competitor containing a mutation in critical residues of the κB binding sites are added (κB mt), this does not compete out the NF-κB band indicating that NF-κB is binding to the radiolabelled wild type probe. (B) EMSA and Competition Assay as described in (A) for cells treated with Thapsigarin (Tg) instead of DTT. Results are similar to the DTT treated cells as described above. (C) Supershift assays identifying the active form of NF-κB contains p65 and p50. Eihter the 5 hr DTT or TG (thapsigargin) samples were incubated with antibodies against p65, p50 or both p65 and p50 for 1 hr. Samples were then run on a gel. Incuabation of p65 resulted in a loss of the NF-κB band, and the appearance of a higher band corresponding to the probe/p65/antibody complex which has a slower mobility on the gel (shifted p65). Incubation with p50 also resulted in disappearance of the NF-κB band and the appearance of a shifted band (shifted p50). Incubation of both p65 and p50 antibodies resulted in disappearance of the NF-κB band and appearance of two shifted bands. This indicates that the activated NF-κB contains p65 and p50. The band labeled (*) could not be shifted by either p65 or p50. (D) Nuclear localization of NF-κB during TG and TNFα treatment. Cells were treated with TG for 1 hr or TNFα for 15 min. Immun [file pone.0045078.s001.tif]

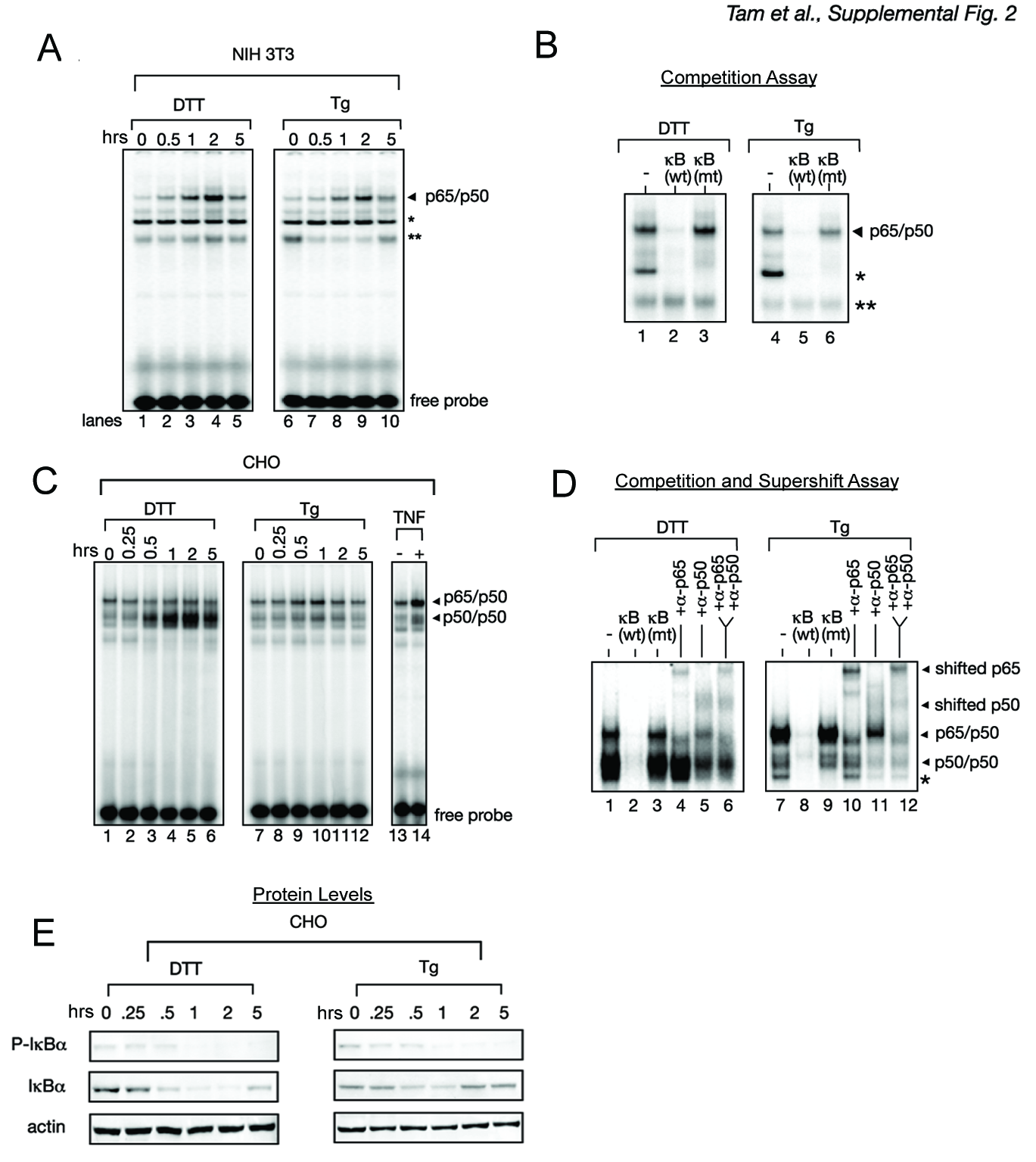

Supplement: Figure S2 — NF-κB activation in multiple cell types: NIH 3T3 and CHO. (A) NF-κB is activated by ER stress in NIH 3T3 cells. NIH 3T3 cells were treated with either DTT or TG for up to 5 hrs, nuclear extracts were prepared, and EMSAs were performed to measure NF-κB activation. NF-κB was activated in treatments with both DTT and TG. (*) and (**) denote background bands as determined by competition assays. (B) Competition assay for NIH 3T3 cells. The 2 hr sample was taken and incubated with a cold oligo containing a wild type or mutant NF-κB binding site. The p65/p50 can be competed out by the wild type competitor but not the mutant competitor demonstrating that the complex observed was p65/p50. Although the band labeled (*) can be competed out by the wild type cold competitor, it can also be competed out by the mutant competitor signifying that it is unlikely to be NF-κB. The band labeled (**) cannot be competed out by the wild type competitor demonstrating that it is a background band. (C) CHO cells were treated with either DTT, TG, or TNFα for up to 5 hrs, nuclear extracts were prepared and EMSAs were performed to measure NF-κB activation. The main form of NF-κB activated by DTT was p50/p50 while TG activated the p65/p50 form of NF-κB as determined by competition and supershift assays in (D). (D) Competition and supershift assays of NF-κB activated in CHO cells. The 2 hr sample was used and incubated with a wild type or mutant cold competitor. Two bands (p65/p50 and p50/p50) were able to be competed by the cold wild type competitor, but were not able to be competed by the cold mutant competitor, indicating that these bands are NF-κB bands. Also, supershift assays reveal the presence of two forms of NF-κB, p65/p50 and p50/p50 as indicated. The p65/p50 band can be shifted by antibodies against both p65 and p50. The p50/p50 band does not shift when only p65 antibody is added, but can be shifted by the p50 antibody, indicating the composistion of the band contains p50 but not p65. ( [file pone.0045078.s002.tif]

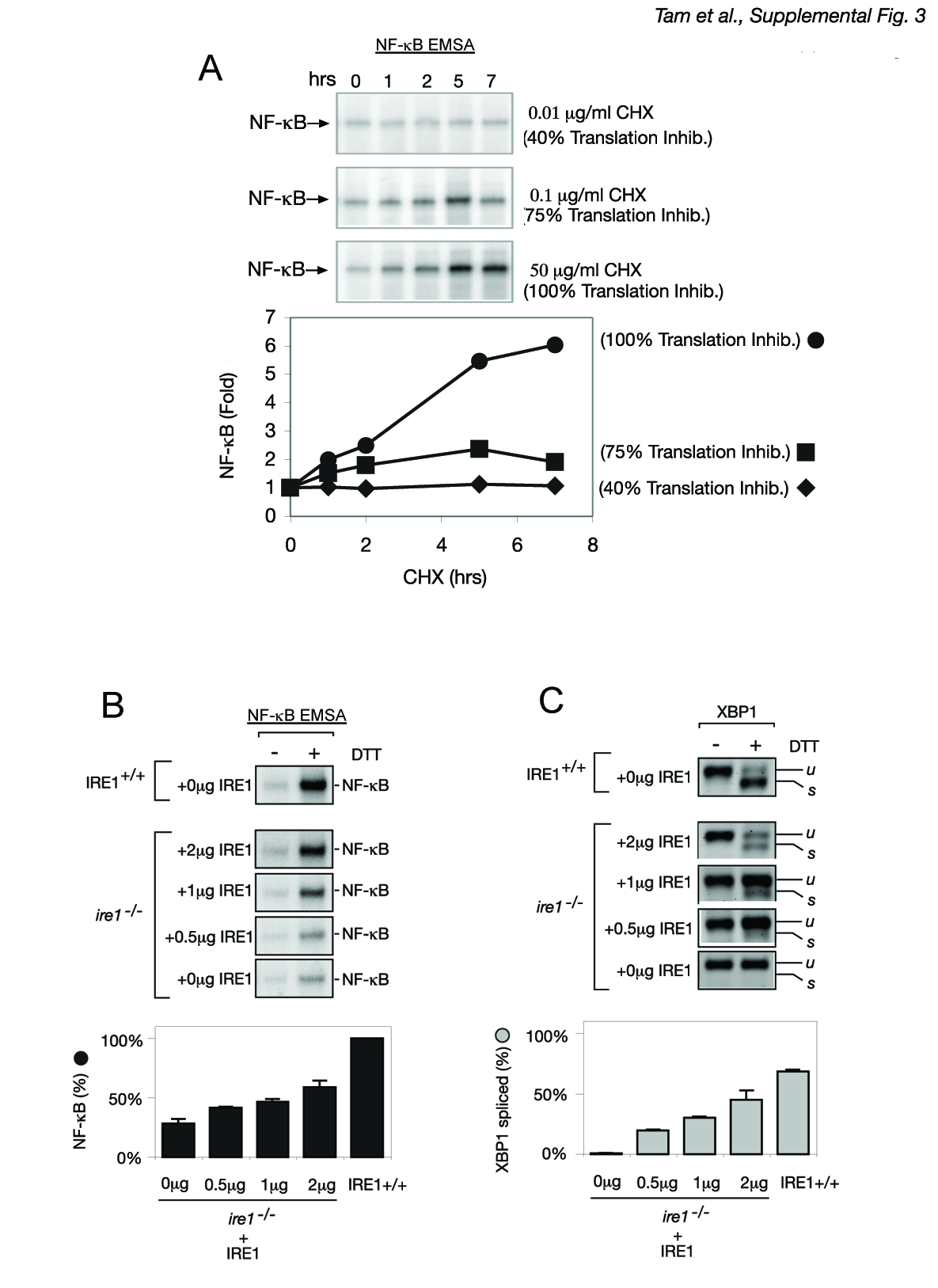

Supplement: Figure S3 — Translation inhibition determines NF-κB activation and addition of IRE1 can rescue NF-κB activation and XBP1 splicing in ire1−/− cells. (A) Degree of translation inhibition determines the strength of NF-κB activation. Increasing concentrations of CHX were used to inhibit translation at different levels. We found that 0.01 µg/ml CHX will inhibit 40% of global translation, 0.1 µg/ml CHX inhibits 75% of global translation, and 50 µg/ml inhibits 100% of global translation using 35S labelling experiments (data not shown). Wild type MEFs were then treated with these concentrations of CHX, and NF-κB activation was determined by EMSA. Increasing the degree of global translation inhibition results in increasing NF-κB activity. 40% translation inhibtion does not increase NF-κB activation significantly. 75% translation inhibition shows a moderate activation of NF-κB. 100% translation inhibition shows the strongest NF-κB activation. (B) Adding back IRE1 can rescue NF-κB activation in ire1 −/− cells. ire1 −/− cells were transfected with increasing amounts of IRE1 plasmid up to 2 µg for 48 hrs. Cells were then treated with DTT for 5 hours, nuclear extracts were prepared, and EMSA assays were performed to determine NF-κB activation in IRE1+/+ or transfected cells during DTT treatment. Transfecting increasing amounts of IRE1 led to increasing activation of NF-κB. Quantitations are shown, and at least three independent experiments were performed. (C) Adding back IRE1 can rescue XBP1 splicing in ire1 −/− cells. RNA was collected from samples in (B) and RT-PCR was performed to determine XBP1 splicing. Unspliced (u) XBP1 contains a 26 nt intron which results in a higher band as compared to the spliced (s) XBP1. ire1 −/− cells are unable to splice XBP1 while WT cells splice XBP1 efficiently during DTT treatment. Adding back IRE1 resulted in increasing rescue of XBP1 splicing in ire1 −/− cells corresponding to increasing rescue of NF-κB activation. (TIF) [file pone.0045078.s003.tif]

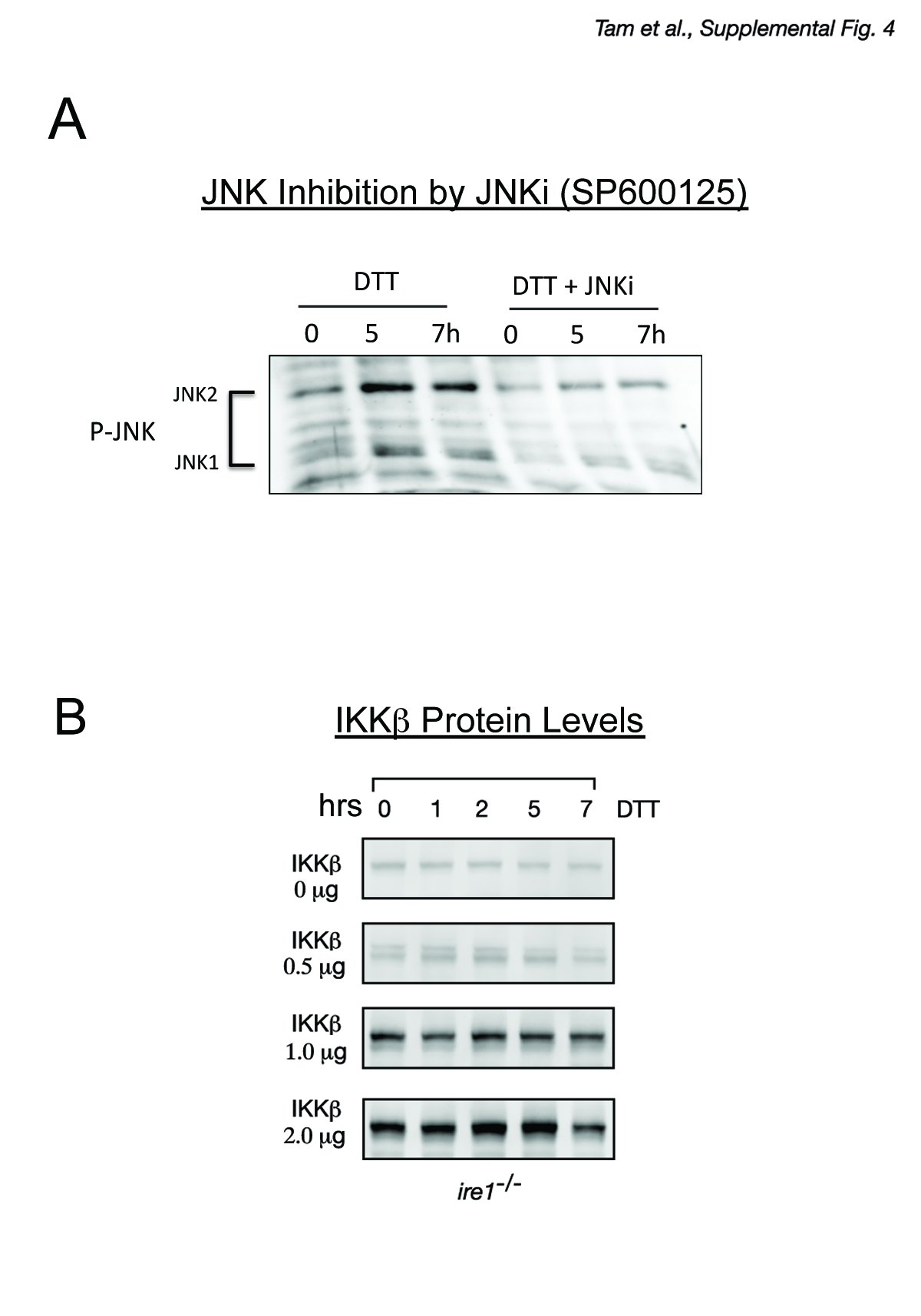

Supplement: Figure S4 — JNK Inhibition and Transfecting IKKβ increases basal IKK activity. (A) JNK inhibitor (JNKi, SP600125) inhibits JNK activation during ER stress. JNKi was added to cells as in Figure 3C. Protein extracts were taken and western blots were performed using antibody detecting active, phosphorylated JNK. Cells treated with DTT showed activation of both JNK1 and JNK2, but when JNKi was added, both JNK1 and JNK2 activation was inhibited. (B) In Figure 4F, transfecting increasing amounts of IKKβ led to increasing basal IKK activity. In order to determine how much IKKβ was transfected, western blots were performed to derermine if equivalent amounts of IKKβ were expressed. Transfecting 0.5 µg plasmid expressed an amount similar to endogenous IKKβ, while 1 and 2 µg expressed significantly more IKKβ. (TIF) [file pone.0045078.s004.tif]
